# Supplementary material for: A fiber array architecture for atom quantum computing
Source: Nat Commun. 2025 Nov 4;16:9728. doi: 10.1038/s41467-025-64738-8 (PMC12586487; doi:10.1038/s41467-025-64738-8)
Supplement: Supplementary file 1 — Supplementary Information [file 41467_2025_64738_MOESM1_ESM.pdf]

Supplementary Information for
"A fiber array architecture for atom quantum computing"

Xiao Li,[1] Jia-Yi Hou,[1,2,*] Jia-Chao Wang,[1,2] Guang-Wei Wang,[1,2] Xiao-Dong He,[1,3] Feng Zhou,[1,3] Yi-Bo Wang,[1] Min Liu,[1] Jin Wang,[1,3,4] Peng Xu,[1,3,†] and Ming-Sheng Zhan[1,3,4,‡]

[1]*Division of Precision Measurement Physics, Wuhan Institute of Physics and Mathematics, Innovation Academy for Precision Measurement Science and Technology, Chinese Academy of Sciences, Wuhan 430071, China*

[2]*School of Physical Sciences, University of Chinese Academy of Sciences, Beijing 100049, China*

[3]*Wuhan Institute of Quantum Technology, Wuhan 430206, China*

[4]*Hefei National Laboratory, Hefei 230088, China*

OPTICAL CONFIGURATION FOR RAMAN LASER BEAMS

In order to independently control 10 qubits, we need 10 sets of Raman laser modules with full control over amplitude, frequency, and phase. We develop a convenient and cost-effective solution, with the optical setup shown in FIG. S1. The two Raman laser beams come from the same 795 nm cat-eye laser. One beam passes through an FEOM for phase modulation, while the other does not. Firstly, the two Raman laser beams are split into ten separate paths. Each pair of split beams is then pass through a single acousto-optic modulator (AOM) from opposite sides, producing +1 order (without FEOM) and -1 order (with FEOM) diffracted beams. After that, the two diffracted beams are combined into a single-mode polarization-maintaining fiber with the same polarization state, forming one pair of addressing Raman laser beams. For this experiment, the FEOM is modulated at 7.054 GHz, and the modulation frequency of the AOM is set around 110 MHz (each AOM's modulation frequency can be finely tuned according to the specific transition frequency of each single-atom qubit). HWP, half-wave plate; QWP, quarter-wave plate; BS, beam splitter.

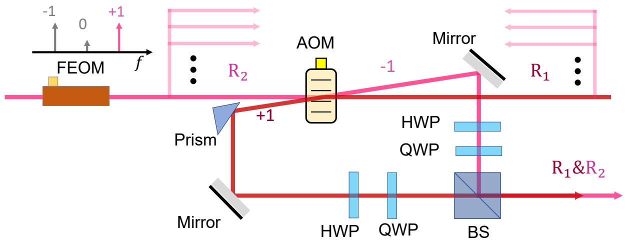

FIG. S1. **Optical setup of Raman laser beams.** Two coherent laser beams, one modulated by an fiber-coupled electro-optic modulator (FEOM) and the other unmodulated, are each split into ten paths. Each pair of split beams (R_1 and R_2) pass through an AOM from opposite sides, obtaining +1 and -1 order diffractions respectively. They are then combined into a single SM PM fiber with identical polarization. The driving frequencies for the FEOM and AOM are set at 7.054 GHz and 100 MHz, respectively. The +1 sideband of the FEOM on R_2, in combination with R_1, forms a pair of Raman beams with a frequency difference of 6.834 GHz.

COHERENCE OF SINGLE-ATOM QUBITS

After optically pumping the single atoms to the state $|0\rangle$, we switch the polarization of the trapping light to σ^+ using an LCVR (Thorlabs LCC1611-B), which stabilizes after 60 ms. With this polarization configuration, single atoms are nearly trapped under magic-intensity conditions, resulting in a significantly enhanced coherence time compared to trapping with linear polarization. Under linear polarization trapping, the T_2^* coherence time of single-atom qubits is around 3.5 ms. However, with magic-intensity trapping, this coherence time significantly increases to over 50 ms [1], as indicated by the Ramsey oscillations in Figure 3 of the main text. However, a tightly focused 795 nm addressing beam (with a waist of 2 μm) will produce a spatially non-uniform differential AC Stark shift on the two encoded hyperfine states, thereby degrading the coherence of the qubits. Ramsey oscillations for one of the qubits, in the presence and absence of the addressing beam, are shown in FIG. S2. Under the influence of the addressing beam, the coherence time of the qubit is reduced to approximately 4.6 ms. We optimize the single-photon detuning Δ for the two-photon Raman transitions by maximizing the product of the coherence time T_2^* and the spin-flip Rabi frequency Ω_R, yielding a value of 200 GHz.

POLARIZATION CONTROL AND ADDRESSING BEAM UNIFORMITY OPTIMIZATION

Different experimental stages impose distinct requirements on the polarization state of the laser beams. During atom loading and polarization gradient cooling (PGC), the trapping light needs to be linearly polarized to achieve low-temperature single atoms. For the site-selective control stage, circular polarization of the addressing light is required to efficiently drive the Raman transitions. We solve this contradiction by dynamically

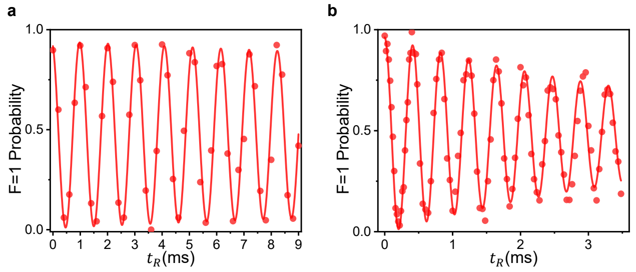

FIG. S2. **Ramsey oscillations for qubit 7 in the absence (a) and presence (b) of the addressing beam.** Under magic-intensity trapping with σ^+ polarization, the coherence time of the qubit exceeds 50 ms. However, under the influence of a tightly focused addressing beam, the coherence time decreases to approximately 4.6 ms.

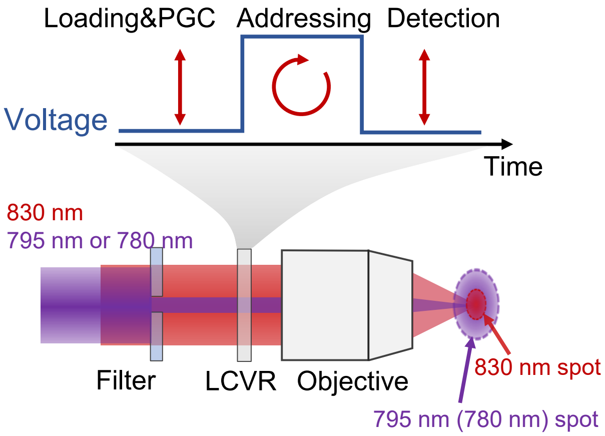

FIG. S3. **Optical methods for switching laser polarization and expanding the addressing spot.** The driving voltage amplitude of the LCVR is varied to meet the laser polarization requirements for different experimental phase. A filter featuring a 1 mm diameter central aperture, which is highly reflective at 795 nm (780 nm) and highly transmissive at 830 nm, is inserted into the optical path to enlarge the beam waist of the addressing spot.

adjusting the laser polarization using a liquid crystal variable retarder (LCVR), as shown in FIG. S3.

Another aspect to take into account is the alignment accuracy between the addressing laser beam spots and the positions of the single atoms, as well as the steep intensity gradient of the tightly focused addressing beams. Both factors affect the spatial uniformity of the addressing light experienced by the atoms. For the first factor, we choose achromatic singlet lenses and customize an apochromatic objective to ensure that the axial chromatic aberration of the optical system is less than 1 μm) from 780 nm to 830 nm range. For the second factor, there are two approaches: lowering the temperature of the single atoms and expanding the waist of the addressing beam spot, the latter being our choice in this work. To increase the addressing beam spot size, as shown in FIG. S3, we insert a filter with a 1 mm diameter central aperture before the dichroic mirror. The addressing laser beam, with an initial diameter of about 4 mm, is reduced to 1 mm after passing through this filter, while the trapping laser beam remains almost unaffected. Consequently, the waist of the addressing beam spot focused on the single atom increases from 0.65 μm to about 2 μm.

While this aperture-integrated filter improves the spatial uniformity of the addressing light experienced by the atoms, it also introduces power loss for the Raman beam. Experimental measurements in our current setup indicate that the 1 mm aperture leads to approximately 80% power loss of the Raman beam. Nevertheless, Raman power utilization can be significantly improved by relaxing the aperture constraint via optimized laser cooling. In our current system, atoms are cooled to approximately 7 μK in a 200 μK trap through a combination of polarization gradient cooling (PGC) and adiabatic cooling, resulting in axial and radial position spreads of approximately 310 nm and 68 nm, respectively [2]. With further implementation of Raman sideband cooling (RSC) [3, 4], the atoms could be cooled to motional ground states, reducing their spatial distributions to 66 nm (axial) and 31 nm (radial). Under these conditions, the aperture diameter can be increased by a factor of 2.5 without compromising coherence or gate fidelity, thereby reducing the Raman beam power loss from 80% to approximately 25%.

OPTICAL CROSSTALK

When individually addressing target qubits, the addressing lasers inevitably impart a small amount of residual intensity onto neighboring spectator qubits, resulting in optical crosstalk. We sequentially measure the Rabi rate crosstalk for each individually addressed single atom, defined by the ratio $\Omega_{\text{adjacent}}/\Omega_{\text{addressed}}$ (where $\Omega_{\text{addressed}}$ and Ω_{adjacent} respectively represent the Rabi frequencies of the addressed and adjacent single atoms). The crosstalk errors are summarized in FIG. S4. Noticeable crosstalk errors are observed between the nearest-neighbor qubits, and they display an asymmetric distribution within the array, mainly due to optical aberrations. The maximum crosstalk, measured at 1.0%, is observed when qubit 4 is addressed, impacting its nearest-neighbor qubit 1.

The maximum observed Rabi rate crosstalk η is approximately 1%, with an average value $\bar{\eta}$ of about 0.2%. Based on the single-qubit gate error model induced by crosstalk [5],

$$\epsilon_\phi = \frac{3}{2}\sin^2\left(\frac{\eta\phi}{2}\right), \tag{S1}$$

we estimate that for a π pulse ($\phi = \pi$), the corresponding

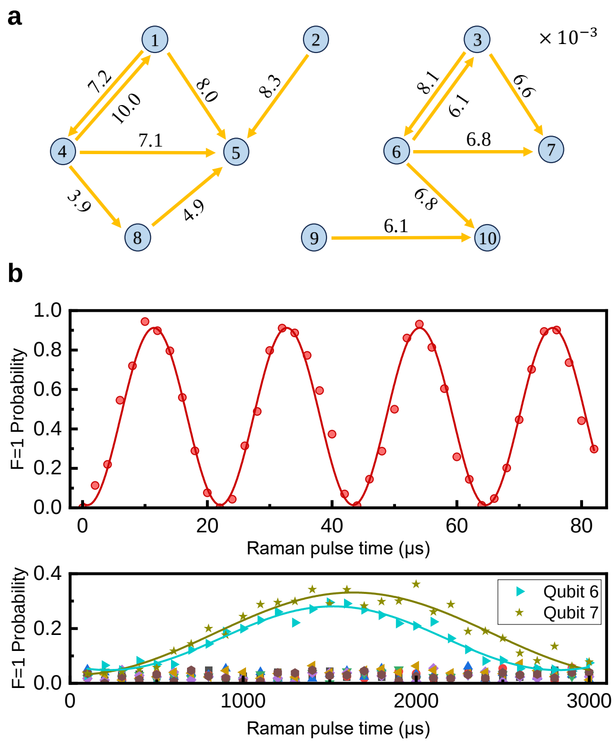

FIG. S4. **Rabi rate crosstalk associated with the individual addressing of each single-atom qubit.** **a**, The overall crosstalk errors across the atom array. Each arrow originates from the addressed qubit and points towards the affected qubit, with the values representing the crosstalk rate. Qubits not linked by an arrow indicate that the crosstalk rate between them is less than 0.1%, similar to what is shown in Figure 2 in the main text, where the longest Raman pulse time used in the experiments is 30 ms. **b**, Example data of crosstalk errors when addressing qubit 3. Driven by the addressing Raman laser, qubit 3 exhibits a Rabi frequency of 47.2 kHz, whereas its neighboring qubits 6 and 7 have Rabi frequencies of 0.38 kHz and 0.31 kHz, respectively. As a result, addressing qubit 3 leads to Rabi rate crosstalk errors of 0.81% for qubit 6 and 0.66% for qubit 7, respectively.

gate errors are approximately 1.6×10^{-4} at maximum crosstalk and 6.6×10^{-6} on average.

In addition to perturbing the quantum evolution of spectator qubits, this crosstalk also degrades the performance of parallel single-qubit gate operations. It originates from spatial interference between addressing beams, leading to shot-to-shot Rabi frequency fluctuations. Addressing beams corresponding to different qubits are transmitted through separate optical fibers. Random disturbances from the room environment inevitably influence these fibers, consequently introducing random phase modulations, $\phi_i(t)$, to the addressing light. This implies that the rate of intensity fluctuations, induced by interference among the addressing beams, depend on the rate of changes in room temperature or air disturbances, which is much slower than the Rabi rate but is comparable to the event-counting rate ($\sim$ 1 Hz in our experiment), and have been confirmed experimentally.

The fluctuation strength depends on the level of crosstalk. A straightforward way to mitigate crosstalk is to reduce the atomic temperature through advanced cooling techniques, which allows approximately a twofold reduction in the addressing beam waist and will significantly reduce spatial overlap between adjacent beams. As also mentioned in [5], there are two feasible strategies to further mitigate this issue: Assign different frequencies to the addressing beams such that the resulting interference occurs at a rate much higher than the Rabi frequency, effectively averaging out the fluctuations; Actively or passively lock the relative phases of the addressing beams to stabilize the interference pattern. The second approach is more suitable for the fiber array architecture. In future work, we plan to explore phase feedback techniques to actively stabilize the phases of the addressing beams [6].

DECOMPOSITION OF CLIFFORD GATE

We decompose the 24 Clifford gate operations into two parts: rotations around axes on the equatorial plane of the Bloch sphere and rotations around the z axis. Supplementary Table 1 displays the specific decompositions of all 24 Clifford gates. The pulse phase is set to 0, π, $\pm\pi/2$, corresponding to the different axes $\pm x, \pm y$ on the equatorial plane, and the pulse area corresponds to the rotation angle around these axes. The $R_z(\theta)$ gates are virtually implemented by offsetting the phase of the addressing laser, which is equivalent to rotating the reference coordinates of subsequent pulses around the z axis by θ.

In the experiment, we employ an Arbitrary Waveform Generator (AWG, Keysight M3301A) and an FPGA processor to implement the set of Clifford gates. Each addressing beam is controlled by an independent AWG channel and a TTL signal from the FPGA. All four AWG channels are employed to demonstrate parallel addressing of arbitrary single atoms. As shown in FIG. S5, the AWG outputs a carrier frequency of 110 MHz RF signal to drive the AOM used for controlling the addressing light. The FPGA processor, in turn, generates programmable TTL signals to control the pulse area of the addressing light. An arbitrary phase offset is realized by the AWG outputting an RF signal distinct from the carrier frequency, while the addressing light keeps off during this period. For example, the AWG outputs an RF signal at 110.5 MHz lasting 0.5 μs, which will produce a π phase offset in the addressing laser. To simplify the process, we keep the phase switching duration fixed and vary the phase shifts by adjusting the frequency difference.

Supplementary Table 1. Decompositions of Clifford gates

Gate Index	$R_x(\theta)$	$R_y(\theta)$	$R_z(\theta)$	U	Pulse Area	Pulse Phase	Phase Offset
0	I	I	I	$\begin{pmatrix}1 & 0\\0 & 1\end{pmatrix}$	0	0	0
1	I	I	$\pi/2$	$e^{-i\pi/4}\begin{pmatrix}1 & 0\\0 & i\end{pmatrix}$	0	0	$\pi/2$
2	I	I	π	$-i\begin{pmatrix}1 & 0\\0 & -1\end{pmatrix}$	0	0	π
3	I	I	$-\pi/2$	$e^{i\pi/4}\begin{pmatrix}1 & 0\\0 & -i\end{pmatrix}$	0	0	$-\pi/2$
4	I	π	I	$-1\begin{pmatrix}0 & 1\\-1 & 0\end{pmatrix}$	π	$\pi/2$	0
5	I	π	$\pi/2$	$-e^{i\pi/4}\begin{pmatrix}0 & 1\\i & 0\end{pmatrix}$	π	0	$\pi/2$
6	π	I	I	$-i\begin{pmatrix}0 & 1\\1 & 0\end{pmatrix}$	π	0	0
7	π	I	$\pi/2$	$e^{-i\pi/4}\begin{pmatrix}0 & 1\\-i & 0\end{pmatrix}$	π	$\pi/2$	$\pi/2$
8	π	$\pi/2$	I	$-\frac{i}{\sqrt{2}}\begin{pmatrix}1 & 1\\1 & -1\end{pmatrix}$	$\pi/2$	$-\pi/2$	π
9	I	$-\pi/2$	I	$\frac{1}{\sqrt{2}}\begin{pmatrix}1 & 1\\-1 & 1\end{pmatrix}$	$\pi/2$	$-\pi/2$	0
10	$\pi/2$	I	$\pi/2$	$\frac{e^{-i\pi/4}}{\sqrt{2}}\begin{pmatrix}1 & 1\\-i & i\end{pmatrix}$	$\pi/2$	$-\pi/2$	$\pi/2$
11	$\pi/2$	π	$\pi/2$	$-\frac{e^{i\pi/4}}{\sqrt{2}}\begin{pmatrix}1 & 1\\i & -i\end{pmatrix}$	$\pi/2$	$-\pi/2$	$-\pi/2$
12	π	$-\pi/2$	I	$\frac{i}{\sqrt{2}}\begin{pmatrix}1 & -1\\-1 & -1\end{pmatrix}$	$\pi/2$	$\pi/2$	π
13	$-\pi/2$	I	$\pi/2$	$\frac{e^{-i\pi/4}}{\sqrt{2}}\begin{pmatrix}1 & -1\\i & i\end{pmatrix}$	$\pi/2$	$\pi/2$	$\pi/2$
14	I	$\pi/2$	I	$\frac{1}{\sqrt{2}}\begin{pmatrix}1 & -1\\1 & 1\end{pmatrix}$	$\pi/2$	$\pi/2$	0
15	$-\pi/2$	π	$\pi/2$	$\frac{e^{i\pi/4}}{\sqrt{2}}\begin{pmatrix}1 & -1\\-i & -i\end{pmatrix}$	$\pi/2$	$\pi/2$	$-\pi/2$
16	$-\pi/2$	$-\pi/2$	I	$\frac{e^{-i\pi/4}}{\sqrt{2}}\begin{pmatrix}1 & i\\-1 & i\end{pmatrix}$	$\pi/2$	π	$\pi/2$
17	$-\pi/2$	$\pi/2$	I	$\frac{e^{i\pi/4}}{\sqrt{2}}\begin{pmatrix}1 & i\\1 & -i\end{pmatrix}$	$\pi/2$	π	$-\pi/2$
18	$-\pi/2$	π	I	$\frac{i}{\sqrt{2}}\begin{pmatrix}1 & i\\-i & -1\end{pmatrix}$	$\pi/2$	π	π
19	$-\pi/2$	I	I	$\frac{1}{\sqrt{2}}\begin{pmatrix}1 & i\\i & 1\end{pmatrix}$	$\pi/2$	π	0
20	$\pi/2$	$-\pi/2$	I	$\frac{e^{i\pi/4}}{\sqrt{2}}\begin{pmatrix}1 & -i\\-1 & -i\end{pmatrix}$	$\pi/2$	0	$-\pi/2$
21	$\pi/2$	I	I	$\frac{1}{\sqrt{2}}\begin{pmatrix}1 & -i\\-i & 1\end{pmatrix}$	$\pi/2$	0	0
22	$\pi/2$	π	I	$\frac{-i}{\sqrt{2}}\begin{pmatrix}1 & -i\\i & -1\end{pmatrix}$	$\pi/2$	0	π
23	$\pi/2$	$\pi/2$	I	$\frac{e^{-i\pi/4}}{\sqrt{2}}\begin{pmatrix}1 & -i\\1 & i\end{pmatrix}$	$\pi/2$	0	$\pi/2$

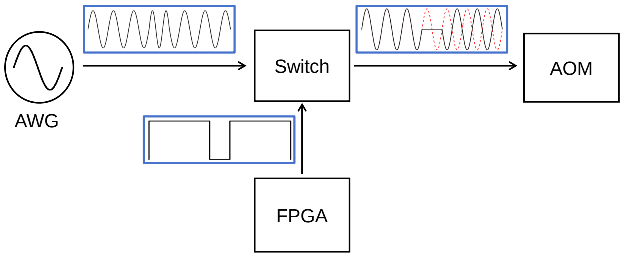

FIG. S5. **The RF chain for controlling a single qubit in the RB experiment.** The AWG outputs a signal to drive the AOM used for controlling the addressing light, and TTL signals control the state flip of the qubit. Phase control of the qubit is achieved by the AWG outputting an RF signal with a frequency that differs from the carrier frequency. As shown by the switch output signals, the solid black line represents the RF signal during phase switching of the qubit, while the red dashed line represents the reference signal without phase switching, indicating a shift in the initial phase of the second RF pulse relative to the first.

* Current address: CAS Cold Atom Technology (Wuhan) Co., Ltd, Wuhan 430074, China
† xupeng@apm.ac.cn
‡ mszhan@apm.ac.cn

[1] Yang, J. et al. Coherence preservation of a single neutral atom qubit transferred between magic-intensity optical traps. *Phys. Rev. Lett.* **117**, 123201 (2016).

[2] Tuchendler, C., Lance, A. M., Browaeys, A., Sortais, Y. R. P. & Grangier, P. Energy distribution and cooling of a single atom in an optical tweezer. *Phys. Rev. A* **78**, 033425 (2008).

[3] Kaufman, A. M., Lester, B. J. & Regal, C. A. Cooling a single atom in an optical tweezer to its quantum ground state. *Phys. Rev. X* **2**, 041014 (2012).

[4] Wang, K. et al. Preparation of a heteronuclear two-atom system in the three-dimensional ground state in an optical tweezer. *Phys. Rev. A* **100**, 063429 (2019).

[5] Radnaev, A. G. et al. A universal neutral-atom quantum computer with individual optical addressing and non-destructive readout. *arXiv:2408.08288* (2024).

[6] Chang, H. et al. First experimental demonstration of coherent beam combining of more than 100 beams. *Photon. Res.* **8**, 1943–1948 (2020).
